# Magnitude, associated factors of difficult airway, and predictive value of airway examinations among maxillofacial surgery patients at public hospitals in Southern Ethiopia: a multicentre cross-sectional study

**DOI:** 10.1097/MS9.0000000000001754

**Published:** 2024-01-26

**Authors:** Abas Ali, Bilen Kassahun, Elias Habtu, Ashebir Debalkie, Kerima Seid, Redi Awol, Mohammed Suleman, Bizuwork Girma, Shamill Eanga, Abdi Oumer, Hassen Mosa, Dawit Tafesse, Temesgen Bati, Getahun Dendir

**Affiliations:** Department ofa Anesthesia, College of Medicine and Health Sciences, Werabe University, Werabe; b Department of Midwifery, College of Medicine and Health Sciences, Werabe University, Werabe; c Department of Anesthesia, Werabe Comprehensive Specialized Hospital, Werabe; d School of Anesthesia, College of Health Sciences and Medicine, Wolaita Sodo University, Wolaita; e School of public health, College of Health Sciences and Medicine, Wolaita Sodo University, Wolaita; f Department of Anesthesia, College of Medicine and Health Sciences, Arsi University, Asella; g Department of Anesthesia, College of Medicine and Health Sciences, Wolkite University, Wolkite; h Department of Anesthesia, College of Medicine and Health Sciences, Selale University, Fitche, Ethiopia

**Keywords:** airway examinations, Associated factors, difficult airway, predictive value

## Abstract

**Background:**

“Difficult airway” is the clinical situation in which a conventionally trained anesthesiologist experiences difficulty with mask ventilation, laryngoscopy, and difficulty with tracheal intubation, or both. Associated factors and predictive value difficult airways, maxillofacial surgery study setup is under studied.

**Objective:**

To determine the magnitude and associated factors of difficult airways and predictive value of airway examinations among adult patients who underwent maxillofacial surgery at public hospitals in Southern Ethiopia 2022.

**Methods and materials:**

An institutional-based multicenter cross-sectional study was conducted among 250 maxillofacial patients from March to 30 June 2022 at selected hospitals. The data were entered into Epi_Data software version 4.3 and analyzed by STATA software version 14. All variables that were statistically significant in bivariate analysis, at the *P* less than 0.25, were included in the multivariate logistic regression analysis. The predictive value or the effectiveness of airway examinations in predicting difficult airways was analyzed by receiver operating curve.

**Results:**

In the current study, the magnitude of difficult airways was 21.2% (95% CI = 16.3–26.1%) and the magnitude of difficult intubation, difficult laryngoscopy, and difficult mask ventilation was 6.4% (95% CI 3.3–9.4%), 9.6% (95% CI 6.1–13%), and 13.6% (95% CI (9.1–16.8%), respectively. History of head and neck surgery adjusted odds ratio (AOR)=6.3, 95% CI (2.85–14.34), cervical collar AOR=4.9, 95% CI (1.96–12.49), and cervical spine injury AOR=2.4, 95% CI (1.07–5.38) were independently and significantly associated with difficulty of airway. Modified Mallampati class and sternomental distance were identified as good preoperative tests to predict difficult laryngoscopy, intubation, and mask ventilation.

**Conclusion and recommendation:**

The magnitude of difficult airways was maxillofacial surgery. Among various airway assessment tests, no single test was perfectly accurate. Anaesthesia professionals are recommended to use a combination of preoperative airway assessments.

## Introduction

Highlights

- A combination of preoperative tests was better for predicting difficult airways.
- Modified Mallampati class and sternomental distance were identified as good preoperative tests to predict difficult laryngoscopy, intubation, and mask ventilation.
- the magnitude of difficult airways was 21.2% among maxillofacial surgery patients.
- history of head and neck surgery and cervical and cervical spine injuries were independently associated with difficulty of airway.
- Among various airway assessment tests, no single test was perfectly accurate.

Airway management is the most difficult aspect of maxillofacial patients and one of the routine activities in anaesthesia practice^1^. Maxillofacial surgery is a type of surgery or operation that focuses on the jaw, mandibular, and facial surgery^2^. With population growth, urbanization, and a changing hurried lifestyle, maxillofacial surgery is becoming a more common component of road traffic accidents (RTAs )^3^.

Anaesthesia for maxillofacial surgery presents several complex issues that are not sharing with other specialties. Patients can present with such a range of abnormalities. Some injuries have minimal impact on the airway, while others cause substantial disruption to the airway anatomy and may necessitate expert airway management^4^. Anaesthetists must pay special attention to the establishment and maintenance of the patient’s airway during these procedures. They must perform a careful upper respiratory tract examination, understand the particular characteristics of certain illnesses, and establish ventilatory access^5,6^.

The most common cause of anaesthesia-related mortality globally is airway issues, which are also the most challenging component of maxillofacial surgery^7^. Worldwide, difficult airway in maxillofacial patients ranged from 11.76 to 32%^8^. In the developed countries, difficult airway is the leading cause of death in maxillofacial patients, with ~600 patients dying each year because of its consequences^9^.

These are far more disastrous in developing countries in which economic problems impart wide varieties of infrastructural challenges such as lack of appropriate facilities and equipment, highly trained anaesthetists, and up-to-date training for the professionals who are part of the client care^10^. In Ethiopia, a study conducted in the general population including maxillofacial surgery showed that the severity of difficult airway during induction of general anaesthesia and the magnitude of difficult laryngoscopy and difficult intubation were 12.3% and 9%, respectively^11^.

Anaesthesia for maxillofacial surgery necessitates careful consideration of the following factors: the nature of the trauma and its effect on the airways, potential difficulties with mask ventilation or endotracheal intubation, possible cervical spine trauma, their limited airways, the risk of regurgitation and aspiration of gastric contents, substantial bleeding that obscures airway anatomy, and the type of surgery that will be performed pose another challenge for anaesthesia providers^12^.

Preoperative evaluation of various anatomical and clinical features aids in the identification of potentially difficult airways. However, the diagnostic accuracy of screening parameters varies between studies due to differences in patient characteristics, type of surgery, ethnicity, gender, and physical and medical characteristics. For example, intubating the trachea in Asian patients may be more difficult than Caucasians^13^.

Early identification of a patient with a suspected difficult airway allows us to plan anaesthetic care and reduce potentially catastrophic consequences^14^. As a result, the purpose of this study was to determine the magnitudes and predictors of difficult airway in adult patients undergoing maxillofacial surgery.

## Methods and materials

### Study design, period and area

A cross-sectional study design was conducted in Wolaita Sodo University Comprehensive Specialized Hospital (WSUCH), Hawassa University Comprehensive Specialized Hospital (HUCSH) and Werabe Comprehensive Specialized Hospital (WCSH). From 1 March to 30 June 2022.The study was registered at www.researchregistry.com with the UIN: research registry 8927. The work has been reported in line with the STROCSS criteria^15^.

### Inclusion criteria

All adult maxillofacial patients who underwent general anaesthesia with endotracheal tube under conventional laryngoscopy and patients who were willing to take part in the study.

### Exclusion criteria

A pregnant mother comes for maxillofacial surgery.
Patients who had goitre came for maxillofacial surgery.

### Operational definitions

- American Society of Anesthesiologists (ASA) classification: Physical status evaluation of the patientshas six classes: I, II, III, IV, V, and VI.
- BMI: Normal weight 18.5–24.9, underweight BMI less than 18.5, and overweight is a BMI greater than 24.9.
- Cervical collar: Material used to stabilize the cervical spine after trauma, surgery, or fractures.
- C-Spine injury: When a bone in the neck (cervical) area of the spine is broken.
- Difficult airways defined as the clinical situation in which a conventionally trained anesthesiologist experiences difficulty with face-mask ventilation of the upper airway, difficulty with tracheal intubation, or both^16^.
- Difficult laryngoscopy: It is not possible to visualize any portion of the vocal cords after multiple attempts at conventional laryngoscopy^17^.
- Difficult tracheal intubation: The appropriate insertion of the endotracheal tube with standard laryngoscopy requires more than three attempts or more than 10 min^18^.
- Difficult face-mask ventilation: When the anaesthetist cannon provide enough ventilation due to improper mask or other device seals, large leakage of gas, or excessive resistance to movement of gas^19^.
- Failed intubation: Inability to intubate the trachea with multiple attempts.

## Study variables

### Dependent variables

- Difficult Airway.

### Independent variables

Sociodemographic variables

- Age, Weight, Height, BMI.
  Preoperative patient-related factors.
- Diabetes history, Mouth opening, ASA status, History of difficulty thyromental distance (TMD), sternomental distance (SMD) mandible protrusion, Mallampati scores.
  Surgery-related factors.
- Indication for surgery, C-Spine injury, cervical collar, Le Fort fracture.

### Sample size determination

The sample size was calculated based on a single population proportion formula assuming the prevalence (P) for the estimated proportion of patients who develop difficulty airway *P*= 0.5 and 5% margin of error at 95% confidence interval using the following formulas:


$$n=\frac{P(1-p)Z{a/2}^{2}}{d^{2}}$$

where *n* = sample size, z= 1.96, *P*= 0.5, w= 0.05, CI=95% & ἀ= 5%


$$\frac{n=\left(1.96\right)2\times 0.5\left(1-0.5\right)=384}{{\left(0.05\right)}^{2}}$$

*nf* = *n*/ (1+*n*/*N*), *N* = 600 (estimated target population) So, *nf* =384 = 234

(384/1+384/600)

By adding 10% of the non-response rate, 234+23=257. Therefore, a total sample size of 257 elective surgical patients was planned to participate in this study.

### Sampling procedures Techniques

A total of four hospitals that provide maxillofacial surgery in the xxx were selected by simple random sampling.

The number of participants was proportionally allocated to each selected hospital based on the average number of expected four-month situational analysis from all hospitals in 2022. Simple random sampling was then used to select study participants.

### Data collection tool and procedure

The questionnaire was developed and adopted after reviewing different literature prepared in English. Orientation on data collection instruments, objectives of the study, and how to facilitate the data collection for data collectors was given by the principal investigator. Data were collected by three trained BSc anaesthetists. Two MSc holder anaesthetists were assigned to supervise the data collection process. After receiving patients into the preoperative waiting area and after giving informed consent, they were assessed by the data collectors on their sociodemographic characteristics. Once the patient entered the operating theatre, data collectors observed and filled out questionnaires for difficult intubation after the induction of general anaesthesia. Intubation was assessed using the ASA guidelines.

### Data quality control

Data quality was assured before, during, and after data collection. Orientation was given for data collectors and supervisors for 2 days on each of the items included in the study tools and the whole process of data collection, objectives, and relevance of the study. During data collection, regular supervision and follow-up were carried out. A supervisor checked each questionnaire daily with a further cross check by the principal investigator for completeness and consistency of data.

### Data processing and analysis

Data were entered in Epi Data 4.3 and then exported to STATA version 14.0 for analysis. Numerical variables were expressed as mean values ± SD, while the other categorical variables were expressed as absolute numbers and percentages. A binary logistic modal was used. The model fit was checked by using the Hosmer-Lemeshow model test with *P* value= 0.3 and multicollinearity of the independent variables was checked by a variance inflation factor (VIF) less than 10 for all independent variables

The χ^2^ test, receiver operating curve (ROC) test, and area under the curve (AUC) was computed. Bivariate analyses were used to identify the candidate variables, which included any variable having *P* less than 0.25 for the multivariate logistic regression analyses model to identify predictors. All variables that were statistically significant in bivariate analysis, at the level of significance of *P* less than 0.25, were included in the multivariate logistic regression analysis. Crude odd ratios and AOR were computed to assess the presence and degree of association between the variables. A *p* value of less than 0.05 was used as statistical significance.

## Results

### Sociodemographic characteristics

A total of 250 study participants were involved in the study, making a response rate of 97.3%.The age range was 18–70 years, with a mean age of 40.9±13.5. The mean BMI of the study participants was 21.1±3.9. Of the total participants, 163 (65.2%) were male and 87 (34.8%) were female (Table 1).

**Table 1:** Sociodemographic characteristics and distribution of difficult laryngoscopy, mask, and intubation among maxillofacial patients

Patient characteristics	Frequency, *n* (%)	DL, *n* (%)	DM, *n* (%)	DI, *n* (%)
Sex				
Male	163	14 (8.6)	18 (11)	9 (5.5)
Female	87	10 (11.5)	16 (18.4)	7 (8)
Age				
18–64	240	22 (9.1)	33 (13.7)	14 (5.8)
>64	10	2 (20)	1 (10)	2 (20)
ASA				
ASA I	161	16 (9.9)	25 (15.5)	10 (6.2)
ASA II	75	6 (8)	6 (8)	5 (6.6)
ASA III	14	2 (14.3)	3 (21.4)	0
BMI				
Normal	222	20 (9)	27 (12.1)	12 (5.4)
Underweight	13	3 (23)	2 (13.4)	4 (26.8)
Overweight	15	0	5 (33.3)	0

ASA, American Society of Anesthesiologists; DI, difficult intubation; DL, difficult laryngoscopy; DM, diabetes mellitus.

### Magnitude of difficult airway

**Figure 1:**
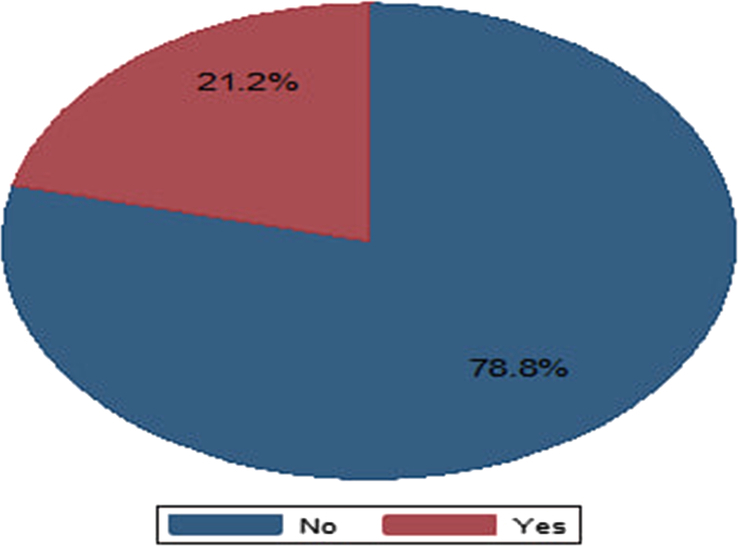
The magnitude of difficult airway in patients undergoing maxillofacial surgery.

The magnitude of difficult airways was 21.2% (95% CI = 16.3–26.1%) (Fig. 1).

### Preoperative airway parameters and their distribution with difficult laryngoscopy, mask and intubation among maxillofacial patients

Maxillofacial patients with inter-incisor distance less than 3 cm had 30 (38.4%) difficult mask ventilation. Also, patients with modified mallampati classes had 20 (15.7%), 25 (19.7%), 13 (10.2%) difficult laryngoscopy, mask, and intubation, respectively. In addition, a patient with thyromental distance less than 6.5 cm had 26 (15%) difficult mask ventilation (Table 2).

**Table 2:** Preoperative airway parameters and their distribution with difficult laryngoscopy, mask, and intubation among maxillofacial patients

Patient characteristics	Frequency, *n* (%)	DL, *n* (%)	DM, *n* (%)	DI, *n* (%)
IID				
<3 cm	78	20 (25.6)	30 (38.4)	11 (14.1)
>3 cm	172	4 (2.3)	4 (2.3)	5 (2.9)
MMC				
Class I and II	64	2 (3.1)	4 (6.2)	1 (1.5)
Class III	59	2 (3.4)	5 (8.4)	2 (3.4)
Class IV	127	20 (15.7)	25 (19.7)	13 (10.2)
TMD				
<6.5 cm	173	18 (10.4)	26 (15)	10 (5.9)
>6.5 cm	77	6 (7.8)	8 (10.4)	6 (7.8)
SMD				
<12.5 cm	104	13 (12.5)	19 (18.3)	8 (7.7)
>12.5 cm	146	11 (7.5)	15 (10.3)	8 (5.5)

ASA, American Society of Anesthesiologists; DI, difficult intubation; DL, difficult laryngoscopy; DM, diabetes mellitus; IID, inter-incisor distance; MMC, modified mallampati classification; SMD, sternomental distance; TMD, thyromental distance.

### Complications in airway management in maxillofacial patients.

**Figure 2:**
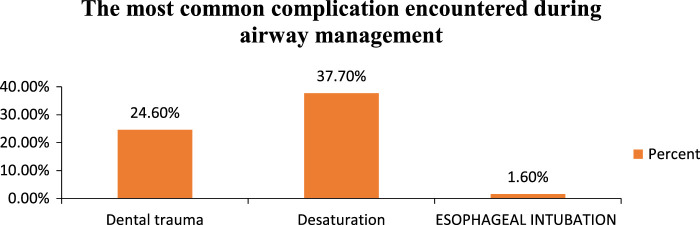
The most common complication encountered during maxillofacial airway management.

The most common complication encountered during airway management is desaturation (37.7%), followed by dental trauma (24.6%) and oesophageal intubation (1.6%)(Fig. 2).

### Factors associated with difficult airways

After controlling confounders Multivariate analysis of the binary logistic model showed that history of head and neck surgery adjusted odds ratio (AOR)=6.3, 95% CI (2.85–14.34), cervical collar AOR=4.9, 95% CI (1.96–12.49), and cervical spine injury AOR=2.4, 95% CI (1.07–5.38) were independently and significantly associated with difficulty of airway (Table 3).

**Table 3:** Factors associated difficult airway among maxillofacial patients

Variables	Frequency	COR (OR (95% CI)	AOR (OR (95% CI)	*P*
C-spine injury				
No	156	1	1	0.032**
Yes	94	4.9 [2.55–9.66]	2.4 [1.07–5.38]	
BMI				
Normal	222	1	1	
Under weight	13	1.1 [1.17–11.49]	2.1 [0.52–9.18]	0.285
Over weight	15	2.1 [0.69–6.59]	2.5 [0.63–10.14]	0.189
Hx of diabetes				
No	243	1	1	0.314
Yes	7	4.9 [1.43–16.75]	2.1 [0.48–9.81]	
Hx of head and neck surgery				
No	204	1	1	**0.001** **
Yes	46	8.5 [4.19–17.33]	6.3 [2.85–14.34]	
Age				
18–64	240	1	1	*
>64	10	1.6 [0.406–6.52]		
ASA				
I	161	1	1	
II	75	0.5 [0.275–1.20]	0.7 [0.28–1.74]	0.444
III	14	1.8 [0.587–5.89]	3.4 [0.95–12.68]	0.059
Cervical collar				
No	222	1	1	0.001**
Yes	28	6.7 [3.14–14.63]	4.9 [1.96–12.49]	

AOR, adjusted odds ratio; ASA, American Society of Anesthesiologists; COR, crude odds ratio.

** Key Significant Association.

### The effectiveness of airway examinations in predicting difficult mask ventilation

**Figure 3:**
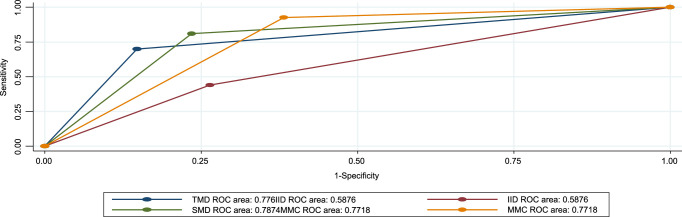
Receiver operating curve (ROC) for preoperative tests against difficult masks shows all tests are above the reference line. IID, inter-incisor distance; MMC, modified mallampati classification; SMD, sternomental distance; TMD, thyromental distance.

**Figure 4:**
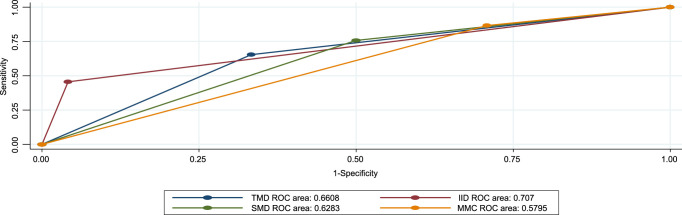
Receiver operating curve (ROC) for preoperative tests against difficult laryngoscopy shows all tests are above the reference line. IID, inter-incisor distance; MMC, modified mallampati classification; SMD, sternomental distance; TMD, thyromental distance.

In this study, modified mallampati classes (MMCs) had a higher sensitivity of 92.6% (95% CI= 89.3–95.8%) and a positive predictive value of 93.9% (95% CI= 90.9–96.8%). SMD had a sensitivity of 81.2% (95% CI= 76.1–85.8%) and a positive predictive value of 95.6 (95%.CI= 93.0–98.1%). The result shows that the SMD had the highest accuracy of 78.7% (95% CI *=* 69.7–85.5). Followed by a TMD of 77.6% (95% CI = 69.7–85.5) (Table 4). All preoperative tests are above the reference line according to the ROC for difficult *mask* tests (Figs. 3 and 4).

**Table 4:** Sensitivity, specificity, positive predictive values, and negative predictive values for difficult mask

							95% CI	
Test	Sn %	SP %	PPV	NPV	*P*	AUC	Lower	Upper
TMD	69.9%	85.3%	96.8%	30.9%	0.000	0.776	0.693	0.851
MMC	92.6%	61.8%	93.9%	56.8%	0.000	0.772	0.670	0.874
SMD	81.2%	76.5%	95.6%	38.8%	0.000	0.787	0.697	0.855
IID	44.0%	73.5%	91.3%	17.1%	0.101	0.588	0.489	0.686

AUC, area under the curve; IID, inter-incisor distance; MMC, modified mallampati classification; NPV, negative predictive value; PPV, positive predictive value; SMD, sternomental distance; Sn, sensitivity; SP, specificity; TMD, thyromental distance.

### The effectiveness of airway examinations in predicting difficult laryngoscopy

**Figure 5:**
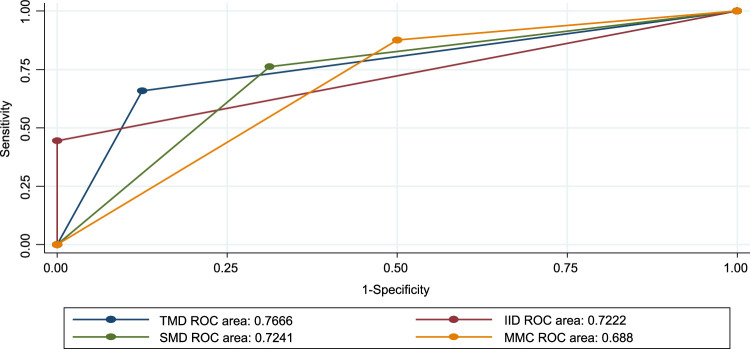
Receiver operating curve (ROC) for preoperative tests against difficult intubation among maxillofacial patients. IID, inter-incisor distance; MMC, modified mallampati classification; SMD, sternomental distance; TMD, thyromental distance.

In this research, the MMCs had a higher sensitivity of 86.7% (95% CI=82.5–90.9%) and positive predictive value 92% (95% CI=88.6–95.3%). SMD also had 75.6% sensitivity and 93.4% positive predictive value. From the table, we can see that inter-incisor distance (IID) showed greater accuracy (70.7%) followed by TMD (66.1%). From the table, we can see that the inter-incisor distance (IID) showed greater accuracy 70.7% (95% CI *=* 65.4–75.9). Followed by TMD (66.1%) (Table 5). All preoperative tests are above the reference line according to the ROC for difficult laryngoscopy tests (Fig. 5).

**Table 5:** Sensitivity, specificity, positive predictive values, and negative predictive values for difficult laryngoscopy

							95% CI	
Test	Sn %	SP %	PPV	NPV	*P*	AUC	Lower	Upper
TMD	65.5%	66.7%	94.9%	17.0%	0.010	0.661	0.559	0.761
MMC	86.7%	29.2%	92.0%	18.9%	0.201	0.579	0.483	0.674
SMD	75.5%	50.0%	93.4%	17.9%	0.039	0.628	0.522	0.734
IID	45.5%	95.8%	99.0%	15.7%	0.001	0.707	0.654	0.759

AUC, area under the curve; IID, inter-incisor distance; MMC, modified mallampati classification; NPV, negative predictive value; PPV, positive predictive value; SMD, sternomental distance; Sn, sensitivity; SP, specificity; TMD, thyromental distance.

### Effectiveness of airway examinations in predicting difficult intubation

**Figure 6:**
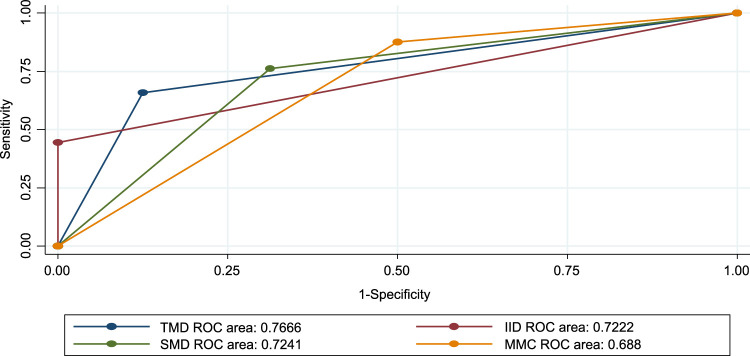
Receiver operating curve (ROC) for preoperative tests against difficult intubation. IID, inter-incisor distance; MMC, modified mallampati classification; SMD, sternomental distance; TMD, thyromental distance.

In this study, MMCs had 87.6% (95% CI= 83.5–91.6%) sensitivity and 96.2% (95% CI= 93.8–98.6%) positive predictive value SMD had a sensitivity of 76.1% (95% CI=71.2–81.7%) and a positive predictive value of 97.3% (95% CI=95.2–99.3%). TMD had the highest accuracy of 76.7% (95% CI = 66.1–87.2), followed by the SMD at 72.4% (95% CI = 58.9–86)(Table 6). All preoperative tests were above the reference line according to the ROC for difficult intubation tests (Fig. 6).

**Table 6:** Sensitivity, specificity, positive predictive values, and negative predictive values for difficult intubation *among* maxillofacial patients

							95% CI	
Test	Sn %	SP %	PPV	NPV	*P* value	AUC	Lower	Upper
TMD	65.8%	87.5%	98.7%	14.9%	0.000	0.767	0.661	0.872
MMC	87.6%	50.0%	96.2%	21.6%	0.012	0.688	0.534	0.842
SMD	76.1%	68.8%	97.3%	16.4%	0.003	0.724	0.589	0.860
IID	44.4%	100%	100%	11.0%	0.003	0.722	0.633	0.812

AUC, area under the curve; IID, inter-incisor distance; MMC, modified mallampati classification; NPV, negative predictive value; PPV, positive predictive value; SMD, sternomental distance; Sn, sensitivity; SP, specificity; TMD, thyromental distance.

## Discussion

In the current study, the magnitude of difficult airways was 21.2% ( 95% CI = 16.3–26.1%) and the magnitude of difficult intubation, difficult laryngoscopy, and difficult mask ventilation was 6.4% (95% CI 3.3–9.4%), 9.6% (95% CI 6.1–13%), and 13.6% (95% CI 9.1–16.8%), respectively. The history of head and neck surgery and cervical and cervical spine injuries were independently and significantly associated with difficulty of airway. MMC and sternomental distance were identified as good preoperative tests to predict difficult laryngoscopy, intubation, and mask ventilation.

In most studies, the degree of mask breathing, laryngoscopy, and intubation difficulty is almost comparable in frequency between the maxillofacial and general surgery populations. This could be because of individual differences in anaesthetic management, including their training, readiness, and access to intubation equipment, which could have an effect on the extent of airway difficulty^20^.

magnitude of difficult laryngoscopy and difficult intubation was consistent with a prior study from Tikur Anbessa^20^, Northern Ethiopia^11^, Nigeria reported that^21^. West Africa found that the magnitude of difficulty laryngoscopy is 3.4%^22^. the breadth of this study is less than ours, which discovered significant variations in the scope of difficulty during laryngoscopy, which have been ascribed to a variety of factors, including cricoid pressure, head position, degree of muscular relaxation, and type or size of laryngoscope blade^22^.

A similar study in India found the magnitude of difficult airway (24.6%)^23^. Another study in China found that the magnitude of difficulties in maxillofacial surgery ranged between 15.4 and 16.9%, which is slightly lower than our findings, possibly due to the use of different terms to define a difficult airway^24^.

Current research patients with cervical collars had five times more difficult airway than non-cervical collars, which is consistent with another similar studies in Nigerian^25^, In Turkey^10^, Korea^26^. The inter-incisor distance’s potential constraints were greatly reduced, and difficulties in visualizing the oral pharynx led to the difficult airway.

Study conducted in Canada^27^, France^28^reduced mobility of those diagnosed with cervical spine limitations are associated factors for difficult intubation. Current study showed that, Patients with cervical spine injury had two times more difficult airway to non-cervical spine injury.

In our studies, inter-incisor distance (IID) and Mallampati score (MMC) predict difficult airway; this result agreed with research findings in Tikur Anbessa Specialized Hospital, wherein the Mallampati test, thyromental distance, sternomental distance, and inter-incisor gap predict difficult airway^29^.

Another study conducted at Gondar University referral hospital in Ethiopia found that the inter-incisor gap and the Mallampati tests are predictors of difficult airway^11^. In related research carried out in Dilla, Ethiopia, sternomental and thyromental distances were found to predict the difficult airway^30^. In studies conducted in West Africa, inter-incisor gap significantly predicted difficult airway *P* values (0.022)^22^. In Indian studies, the inter-incisor gap and the Mallampati tests were found to be independent predictors of difficult airways^13^.

A similar study was conducted in India, and the SMD, TMD, and Mallampati tests predicted difficulty in the airway, which agrees with our findings^31^. Our research found that SMD, TMD, IID, and MMC have good sensitivity, specificity, and positive predictive values but poor negative predictive values. Comparable results were obtained in similar studies^20,32^. Another comparable study was conducted in Tikur Anbessa Specialized Hospital, Ethiopia, which identified MMC and IID as independent predictors of difficult laryngoscopy and good sensitivity, specificity, and negative predictive values but poor positive predictive values for TMD, MMC, SMD, and IID^32^. The reason for poor positive predictive values could be due to interpersonnel differences in anaesthesia management, including preparation, experience, and availability of equipment.

Similar research was conducted in northern Ethiopia, which agrees with our studies, where they were found to have good sensitivity and specificity values for TMD, MMC, SMD, and IID^11^. The reason for good negative predictive values may be the result of interpersonnel variations in anaesthesia management.

Further, the current study findings were in line with those found in Jimma, Ethiopia. The sensitivity, specificity, PPV, and NPV of difficult intubation were as follows: [100%, 96.64%, 20%, and 100%] for TMD; and [100%, 99.14%, 75%, and 100%] for the Mallampati test^29^. In contrast to the above findings, a study conducted in West Africashows poor sensitivity but better specificity^22^. The reasons for poor sensitivity and positive predictive values may be due to differences in patients’ physical appearance, sample size, and cutoff values for the screening tests. Similar results were obtained in a study conducted in Turkey, where it was found that MMCs, TMDs, SMDs, and IIDs had the following sensitivity, specificity, and positive and negative predictive values of difficulty intubation: (59%; 28%;31%; 31%), (83%; 88%; 90%; 96%), (39%; 30%; 37%; 58%)and (92%; 87%; 88%; 88%)^33^.

A combination of several parameters could result in improved prediction performance. These assessments often incorporate various variables into a scoring system to anticipate difficult intubation^13,34,35^. consistently, current studies indicate the effectiveness of airway examinations in predicting difficult intubation All preoperative tests are above the reference line according to the receiver operating curve for difficult intubation tests. AS result using different airway examinations is valuable to predict difficult intubation.

## Conclusions

In the current study, the magnitude of difficult airways was 21.2% and the magnitude of difficult intubation, difficult laryngoscopy, and difficult mask ventilation was 6.4%, 9.6%, 13.6%, respectively. The history of head and neck surgery and cervical and cervical spine injuries were independently and significantly associated with difficulty of airway. Modified Mallampati class and sternomental distance were identified as good preoperative tests to predict difficult laryngoscopy, intubation, and mask ventilatio. A combination of preoperative tests was identified as being better for predicting difficult airways than individual preoperative tests. Therefore, every anaesthesia professional is recommended to use a combination of preoperative airway assessments.

## Ethics approval and consent to participants

Ethical approval was obtained from Institutional Review board of Wolaita Sodo University, College of Health Sciences and Medicine, with protocol unique No. CHSM/ERC/03/14 and submitted to wolaita sodo comprehensive specialized hospital. Then, permission was obtained from the hospitals to conduct the study. Informed consent was taken from every study participant. Informed consent was taken from every study participant; study is done according to Helsinki Declaration

## Consent for publication

Not applicable.

## Source of funding

Wolaita Sodo University funded this study, and it is open for researchers to publish the manuscript. The funder played no role in the study design, data collection, analysis, decision to publish, or preparation of the manuscript.

## Author contribution

A.A. and G.D. were involved in the conception, study design, execution, acquisition of data, analysis and interpretation of data, and took part in drafting the article or revising it critically for important intellectual content. B.K., E.H., A.D., K.S., R.A., M.S., B.G., S.E., A.O., H.M., D.T. and T.B. were involved in study design, execution, and acquisition of data, interpretation, drafting and final manuscript writing.

## Conflicts of interest disclosure

The authors declare that they have no competing interests.

## Research registration unique identifying number (UIN)

The study was registered at www.researchregistry.com with the UIN: research registry 8927.

## Guarantor

Getahun Dendir and Abas Ali.

## The availability of data and materials

The data that support the findings of this study are available from the corresponding authors upon reasonable request.

## Provenance and peer review

Not commissioned, externally peer-reviewed.

## Acknowledgements

First of all, I would like to thank Wolaita Sodo University, College of Health Science and Medicine, School of Anesthesia, for giving me this opportunity to develop a thesis project. My thanks would also extend to all my friends and Wolaita Sodo University Comprehensive Specialized Hospital (WSUCH), Hawassa University Comprehensive Specialized Hospital (HUCSH) and Werabe Comprehensive Specialized Hospital (WCSH) staff for their unlimited support for the success of this research project.
